# Health effects of plants, light, and natural elements of biophilic interventions in confined settings: a systematic review

**DOI:** 10.3389/fphys.2025.1700518

**Published:** 2025-12-08

**Authors:** Abdolrahim Zandi, Shu-Fen Wung

**Affiliations:** 1 University of North Dakota, Department of Biomedical Engineering, Grand Forks, ND, United States; 2 University of California Davis, Betty Irene Moore School of Nursing, Davis, CA, United States

**Keywords:** biophilic intervention, isolated habitat, body and mind care, artificial biosphere, attention restoration theory (ART), stress reduction theory (SRT)

## Abstract

Biophilic intervention strategies that incorporate plants, light, and organic elements are increasingly recognized for supporting well-being in confined environments. This systematic review analyzes health outcomes associated with edible greens and biophilic elements across 124 studies drawn from PubMed and Scopus, following PRISMA guidelines. The evidence demonstrates that greenery in confined settings—such as hospitals, eldercare, and space habitats—reduces stress, improves mood, and accelerates recovery, consistent with Stress Reduction Theory (SRT) and Attention Restoration Theory (ART). In space analogs, plant-based modules support cognitive function and improve habitat experience by producing food and oxygen. Despite these benefits, a few challenges remain: infection control, spatial constraints, and operational limitations can hinder adoption. Nonetheless, tailored biophilic systems represent a promising path to enhance health and resilience in both terrestrial and space-based care environments. This review synthesizes findings from both terrestrial and extraterrestrial environments to evaluate the effectiveness of edible plant-based biophilic interventions. Evidence from clinical studies and long-duration missions suggests that incorporating edible vegetation into confined environments enhances psychological resilience, supports nutritional intake, and contributes to overall well-being. The presence of living plant systems has been shown to reduce stress, enhance mood, and foster a sense of connectedness to nature in contexts where natural stimuli are otherwise absent. Together, these results support the role of edible greens as practical, scalable components for designing sustainable, health-promoting environments in both Earth-based and space-based habitats. We examined the role of biophilic interventions, particularly the incorporation of edible greens, in promoting health within confined environments. Biophilic interventions incorporate natural forms, materials, edible plants, and natural light into architectural designs and indoor settings to enhance both physical and mental well-being (Body and Mind Care). Research in clinical settings and space missions has focused on the outcomes associated with human-plant interactions and the development of bio-regenerative plant modules that support sustainable living. These systems grow plants in controlled environments, enabling food production and the regeneration of essential life-support resources, such as oxygen and clean air. They aim to support crew health through food production, air purification, and psychological benefits, particularly during long-duration missions. We conducted a systematic review, searching databases including PubMed and Scopus, and selected 124 studies based on the PRISMA criteria to analyze the impact of these interventions in eldercare, hospitals, isolation-wards, and spaceflight. Incorporating natural elements into confined habitats yields notable psychological and physiological benefits. In healthcare and indoor environments, the presence of greenery consistently reduces stress, elevates mood, and improves patients’ perception of their surroundings, often contributing to faster recovery. These effects are not limited to hospitals and eldercare settings. In remote and extreme environments, such as polar research stations and space missions, plant interaction can alleviate cognitive fatigue, reduce monotony, and strengthen team cohesion. Integrating edible greens and biophilic elements into confined settings—such as hospitals, eldercare facilities, and space habitats—offers measurable benefits for psychological resilience, reduced physiological stress, and improved cognitive performance. These systems serve dual purposes: therapeutic exposure to nature and support for nutritional or regenerative goals. In hospitals and long-term care, interventions like healing gardens or nature-themed spaces have been shown to reduce anxiety, pain perception, and cortisol levels, while enhancing mood and focus (Beukeboom et.al., 2012; Detweiler et al., 2012). However, high-risk environments like ICUs and operating rooms face practical barriers, including infection control, equipment sensitivity, and space limitations. Similarly, in analog and orbital habitats such as HERA or the ISS, biophilic integration is constrained by power, volume, microbial safety, and crew workload. Despite these constraints, evidence supports the feasibility of modular, low-risk systems—including sealed plant modules, artificial daylighting, and virtual green exposure—tailored to operational demands. As confined living environments become more common across clinical and off-world contexts, biophilic strategies present an adaptable, scalable framework for enhancing well-being, with minimal disruption to safety or efficiency.

## 1 Background

Psychological resilience in confined, remote, and highly controlled environments—such as space habitats, polar research stations, field hospitals, and long-term care units—is increasingly recognized as essential to operational performance and recovery. These environments often restrict access to daylight, vegetation, and natural airflow due to safety, sterility, or spatial constraints. While life-support systems typically prioritize oxygen, food, and waste recycling, they often neglect the emotional and sensory benefits provided by plant life (Odeh and Guy, 2017). In critical medical contexts like ICUs and disaster-response shelters, live vegetation is usually excluded due to infection control and maintenance limitations. Still, even limited exposure to plant imagery or natural light has been associated with improved patient mood, enhanced caregiver focus, and reduced stress in mobile hospitals and post-disaster clinics (Beukeboom et.al., 2012; Lohr and Pearson-Mims, 2008).

In spaceflight settings, astronauts have reported emotional attachment to plant-growth modules like NASA’s Veggie system. These systems serve not only as food and oxygen sources, but also as calming, interactive stimuli during high-stress mission phases (Haeuplik-Meusburger et al., 2014). Comparable benefits have been documented in Antarctic stations, where indoor plant chambers improved sleep, cognitive clarity, and group cohesion (Wood et al., 2022; Massa G. D. et al., 2017). Plants’ multisensory presence—via scent, color, and tactile interaction—serves as a potent countermeasure to sensory monotony. When integrated into modular life-support or medical systems, they offer measurable benefits in psychological stabilization, autonomic recovery, and overall resilience (Park and Mattson, 2009a).

### 1.1 Psychological and nutritional value of edible greens in confined settings

Indoor plants have well-established benefits for patient outcomes. In a landmark study, surgical patients with tree-view windows experienced shorter hospital stays, fewer nurse-reported complications, and reduced pain medication compared to those facing a brick wall (Gaugler, 2005). Similar advantages emerge in isolated environments—residents at Antarctic stations, who interact with greenhouse plants, report enhanced psychological resilience and reduced monotony. Analog missions such as NASA’s HERA further confirm that plant interaction supports cognitive focus and emotional stability (Friedmann et al., 2015; Sandal et al., 2018).

### 1.2 Acute care settings: hospitals and intensive care units (ICUs)

At the Royal Brisbane and Women’s Hospital, the NICU redesign introduced calming biophilic elements, including soft lighting, nature-inspired artwork, and an intuitive way-finding system. Although participant statistics were not reported, staff noted improvements in orientation, reduced stress, and a more nurturing atmosphere, without disruption to clinical operations (Speicher and Francis, 2023; Wright et al., 2017).

### 1.3 Long-term care facilities

Eldercare providers have integrated indoor plants, therapeutic gardens, imagery, and natural light to enhance mood, cognitive engagement, and blood pressure regulation, particularly for residents with dementia or chronic illnesses (Detweiler, 2012). These interventions align with SRT and ART, which link nature exposure to stress reduction and cognitive restoration (Ulrich, 1984; Kaplan and Kaplan, 1989). However, care must be taken to avoid allergen or microbial risks, particularly in understaffed facilities (Khan, 2023; Sholanke and Eleagu, 2024).

### 1.4 Psychosocial support in analog space missions

Biophilic modules in analog environments (HERA, NEEMO, Concordia Station) consistently report mood improvement, enhanced focus, and reduced stress among crew members (Keng et al., 2011; Friedman, 2002). These controlled settings validate plant-based strategies before deployment in space.

### 1.5 In-orbit and extraterrestrial habitats

Onboard ISS, systems like VEGGIE and Advanced Plant Habitat support astronauts both nutritionally and psychologically, offering sensory engagement and emotional grounding (Massa F. G. et al., 2017; McGreevy and Boland, 2020). Moving forward, scalable, sterile-compatible biophilic modules will be essential for future lunar or Martian habitats.

## 2 Review methodology and strategy

**FIGURE 1:**
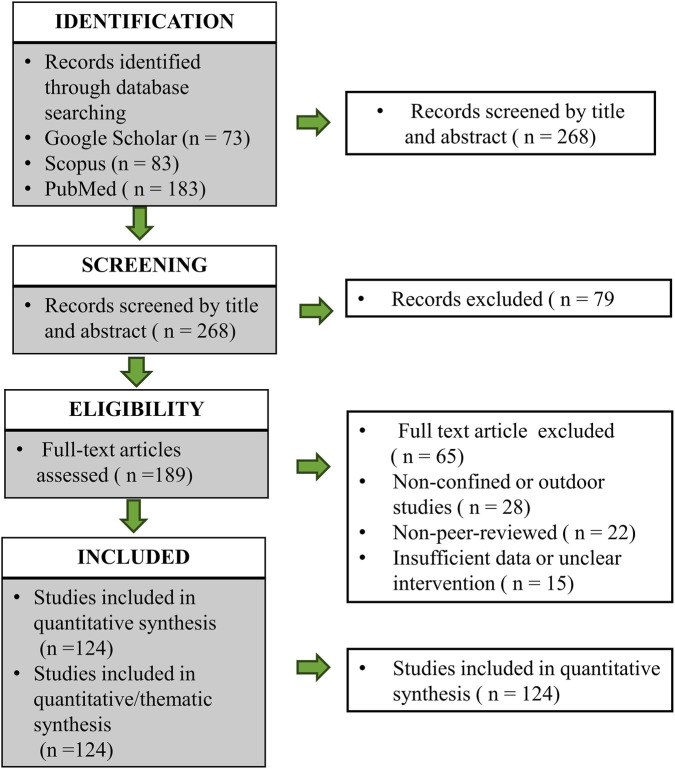
Flow diagram for new systematic reviews.

We conducted a comprehensive literature search across Google Scholar (473 records), Scopus (93 records), and PubMed (183 records), resulting in 749 records, to identify studies published between 2000 and 2025 that focus on biophilic interventions in confined environments. The keywords used in the search included various combinations of “biophilic,” “greens,” “health,” “space,” “habitats,” and “intervention.” After removing duplicates, we screened a total of 268 unique records, and from these, we evaluated 189 abstracts and selected 124 full-text studies for detailed analysis. Our review’s inclusion criteria were peer-reviewed studies involving human or human-analog environments (e.g., hospitals, space modules, and long-term care facilities) that included interventions involving plants or green systems. Exclusion criteria included non-biological interventions and studies conducted in non-confined terrestrial environments. The methodology adhered to PRISMA guidelines to ensure transparency, replicability, and analytical rigor. Figure 1 illustrates the systematic review flowchart that evaluates biophilic design outcomes in healthcare and space contexts. This systematic review was structured according to the PICOS framework to ensure clarity and reproducibility. Population: individuals living or working in confined, clinical, or isolated environments (e.g., hospitals, eldercare facilities, space analogs, and orbital habitats). Intervention: biophilic design elements such as edible greens, indoor vegetation, natural light, and nature-inspired materials. Comparator: conventional or non-biophilic environments when available. Outcomes: physiological, psychological, and behavioral indicators of well-being. Study design: peer-reviewed observational studies, clinical trials, and controlled analog experiments meeting inclusion criteria. All figures were prepared in high-resolution (≥300 dpi) or vector format, and all references were cross-checked for accuracy, relevance, and correct attribution prior to resubmission.

## 3 Theoretical foundations of biophilic design

Biophilic design intentionally incorporates elements like vegetation, daylight, water, and natural textures into built environments to support well-being. It is grounded in established frameworks such as Stress Reduction Theory, which posits that exposure to nature lowers physiological stress (e.g., cortisol, blood pressure), and Attention Restoration Theory, which holds that natural settings help replenish cognitive focus. Foundational studies have demonstrated that human interaction with natural features supports physical, emotional, and cognitive health across settings (Zhong et al., 2022; Grinde and Patil, 2009; Joye and De Block, 2011), forming the theoretical basis for biophilic integration in healthcare and space design.

### 3.1 Historical and environmental context

**FIGURE 2:**
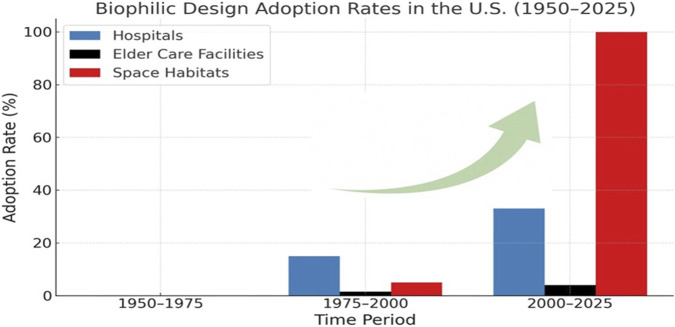
Biophilic design adaptation Rates (1950–2025). The term “biophilia” was coined in 1984, well after 1950s United States hospitals, which followed a sterile, minimalist International Style focused on efficiency and infection control, with little regard for nature or patient-centered design.

Confined environments—such as ICUs, eldercare facilities, spacecraft, and analog stations—often lack daylight and sensory variability, contributing to psychological strain and cognitive fatigue (Lerer and Varia, 2022; Basner, 2021). In response, designers in healthcare and aerospace have adopted biophilic strategies. Since Ulrich’s landmark 1984 study demonstrated that natural views improve patient recovery, hospitals have introduced healing gardens and daylight access. Space analogs like the ISS and Concordia Station now incorporate plant-growth modules to mitigate the psychological toll of isolation (Haeuplik-Meusburger et al., 2014). These strategies reflect a shift from utilitarian 1950s models to sensory-rich, human-centered systems in the 2020s (Montañana et al., 2024; Gushin, 2021) (see Table 1 and Figure 2).

**TABLE 1:** Biophilic design adoption rates (1950–2024).

Period	Hospitals	Elder-care facilities	Space habitats/analogs	References
1950–1975	∼0%; design dominated by sterile, functional aesthetics; virtually no biophilic features	∼0%; institutional layouts with minimal greenery or natural elements	0 — Early missions (Mercury/Gemini/Apollo), Skylab had only a viewing window; no biophilic systems	Ulrich (1984), Petros and Georgi (2011), Compton et al. (2011)
1975–2000	∼10–20% — some hospitals added healing gardens and nature-based art following Ulrich’s work	∼200 facilities (∼1–2%) adopted the Eden Alternative approach by 1999	Low (<5%); Shuttle/Mir plant trials; Biosphere 2 analog, wheat experiments for life support	Ulrich (1984), Marcus and Barnes (1995), Thomas and Xing (2021), Ferl et al. (2002)
2000–2025	∼33% designated healing gardens; ∼33–40% integrated nature access/views; ∼50% had art-nature programs by early 2010s	∼300 Eden homes +382 Green House homes (∼4% of ∼16,100 U.S. facilities as of 2023)	High (∼100%); ISS with Cupola window and Veggie plant systems since 2010; analog habitats include biophilic elements	Kent (2015), Tracada (2024), Firth and Jayadas (2022), Kubsch et al. (2018), Zeidler et al. (2017), Massa G. D. et al. (2017)

### 3.2 Biophilic integration and human resilience

Incorporating edible greens and natural stimuli into enclosed environments enhances resilience and sustainability. Biophilic design has demonstrated tangible benefits across sectors, including healthcare and workplace settings. For instance, it has been linked to increased well-being (47%), creativity (45%), and productivity (38%) in office contexts (Zhong et al., 2022). In hospitals, it reduces hospitalization time, pain perception, and stress among both patients and providers (Díaz Díez, 2024).

### 3.3 Cognitive and physiological effects

Biophilic environments improve cognitive function and stress regulation. Multimodal biophilic design patterns yield greater physiological benefits than isolated elements (Zhang et al., 2024). Observed outcomes include reduced cortisol, improved HRV, enhanced attention and mood, and higher post-occupancy satisfaction (Aristizabal, 2021). While causal links to longevity are complex, exposure to nature is consistently associated with reduced all-cause mortality and better quality-adjusted life years (James et al., 2016).

### 3.4 Controlled environments as unique stressors

Spaces like submarines, polar stations, and ICUs present unique challenges—confinement, isolation, circadian disruption, and reduced agency. These settings often lack varied sensory input and autonomy, increasing vulnerability to anxiety, cognitive decline, and depressive symptoms (Basner, 2021; Feuerecker et al., 2019). Disrupted circadian rhythms, common in artificial lighting environments, further impair sleep, cognition, and hormone balance (Wyatt et al., 2025).

Environmental deprivation dulls attention and decision-making (Mammarella, 2021),while cumulative stressors may impair neuroendocrine and immune systems (Stahn and Kühn, 2021). As countermeasures, biophilic design, artificial circadian lighting, and multisensory stimulation are being implemented to improve outcomes in closed environments (Jung et al., 2023).

Figure 2 summarizes the historical adoption of biophilic design across hospitals, eldercare settings, and space environments. “Adoption rate” refers to the proportion of facilities incorporating at least one biophilic element—such as vegetation, daylight, or nature-inspired features—into their physical environment. In the 1950s, such environments were largely utilitarian. A pivotal shift occurred after Ulrich (1984) study, which linked nature views to faster recovery and reduced analgesic use(Ulrich, 1984). Over the next decades, hospitals and eldercare centers began integrating features like healing gardens and daylight exposure. This trend accelerated in the 2020s as further evidence demonstrated benefits for both patient outcomes and staff wellbeing (Montañana et al., 2024). Eldercare environments similarly evolved from low-stimulation interiors to designs that incorporate sensory gardens and indoor greenery supporting cognitive and emotional resilience (Gushin, 2021).

## 4 Edible plants as dual-purpose interventions

### 4.1 Nutritional values

Microgreens, such as kale, radish, and mustard greens, are rich in essential nutrients and bioactive compounds. Microgreens are young, edible seedlings of vegetables and herbs harvested at the cotyledon stage or when they first develop true leaves, typically 7–21 days after germination. They are more mature than sprouts, harvested earlier than baby greens, and known for their concentrated nutrients, intense flavors, and vibrant colors (Xiao et al., 2012). They contain high levels of vitamins C, E, and K, as well as minerals such as iron and magnesium. Additionally, these plants are rich in antioxidants, including phenolic compounds and carotenoids (Marques et al., 2021). These nutrients support immune function, reduce oxidative stress, and promote overall health. Studies have shown that microgreens can offer significantly higher nutrient densities than their mature counterparts, making them a valuable addition to diets, especially in controlled environments where nutritional variety may be limited (Bhaswant et al., 2023; Ayeni, 2021).

### 4.2 Psychosocial benefits

Engaging in horticultural activities, such as growing and consuming edible plants, has been associated with enhanced psychological wellbeing. Horticultural therapy has reduced stress, anxiety, and depression while also improving mood and social interactions (Soga et al., 2017; Detweiler, 2012). These benefits are particularly significant in isolated or confined settings, where interaction with nature and engaging in purposeful activities can help alleviate the adverse effects of isolation and monotony. Caring for plants fosters a sense of responsibility and accomplishment, which contributes to improved mental health outcomes (Ainamani et al., 2022; Dijkstra et al., 2008). found that indoor plants in hospital waiting rooms helped reduce patients’ perceived stress. Their study suggested that this stress-reducing effect was mediated by increased environmental attractiveness, thereby confirming the importance of biophilic design in indoor healthcare settings (Dijkstra et al., 2008).

## 5 Evidence synthesis from clinical and confined settings

To better understand the impacts of biophilic and plant-based interventions across various confined settings, we synthesized evidence from clinical and space-analog environments. The following subsections present selected case studies and experimental findings from hospital intensive care units, long-term care facilities, and isolation environments. These examples highlight both the benefits and limitations of implementing biophilic strategies in settings where infection control, spatial constraints, and psychological stress are critical factors.

### 5.1 Quantitative overview of physiological effects

To complement the qualitative synthesis, we identified studies reporting statistically robust physiological outcomes from biophilic interventions in confined environments. Table 2 summarizes a subset of these studies, highlighting key metrics, including sample size, intervention type, outcome variables (e.g., cortisol, blood pressure, EEG indices), and reported statistical significance. Where available, mean values, standard deviations, and p-values are included to illustrate the strength and consistency of effects. These findings underscore the measurable physiological benefits of exposure to nature-based elements in clinical or isolated settings.

**TABLE 2:** Quantitative physiological outcomes from biophilic intervention studies.

Study	N	Outcome measured	Pre mean ± SD	Post mean ± SD	Test used	p-value	Effect size/notes
Aristizabal (2021)	35	NS-SCR Frequency (per min)	Baseline: ∼1.55	Visual: ↓1.55, Multi: ↓2.65	Mixed-effects model	<0.001	Significant reductions in both conditions
		NS-SCR Amplitude (μS)	0.24 (est.)	↓0.79 (multi vs. base)	Mixed-effects model	0.007	CI: −0.22 to −0.03
Yin et al. (2020)	100	Diastolic BP (mmHg)	–	↓4.5 (Indoor nature)	ANOVA	<0.05	Greater reduction than control
		HRV Recovery (RMSSD)	–	+2.1%/min improvement	Linear regression	∼0.05	Faster recovery rate
Kari et al. (2024)	39	Heart Rate (bpm)	73.6 ± 10.1	71.8 ± 9.8	Repeated Measures ANOVA	<0.05	Nature break yielded greater HR reduction
Toyoda et al. (2020)	63	Trait Anxiety (STAI)	39.0 ± 9.1	36.5 ± 8.7	Paired t-test	<0.05	Cohen’s d ≈ 0.27 (small effect)
		Pulse Rate Reduction (binary)	14% (baseline)	27% (with plant)	χ^2^ test	<0.05	More participants had dropped with the plant
Douglas et al. (2022)	413	Skin Conductance (SCR, μS)	0.038 (artificial)	0.021 (natural)	2 × 2 ANOVA	0.023	η^2^ ≈ 0.01; CI: −0.013 to −0.00029

HR: heart rate; HRV: heart rate variability; BP: blood pressure; GSR: Galvanic Skin Response (also known as SCR); SCR: skin conductance response; EEG: electroencephalography; fNIRS: Functional Near-Infrared Spectroscopy; SpO_2_: blood oxygen saturation; NS: not significant; NCR: no change reported.

### 5.2 Hospital and ICU-Based studies

Clinical studies have demonstrated that integrating natural elements, such as aromatherapy and access to a green environment, into hospital and intensive care unit (ICU) settings can significantly enhance patient wellbeing. For instance, a randomized controlled trial found that lavender aromatherapy improved sleep quality and reduced anxiety among 60 patients in a coronary intensive care unit (ICU) (Karadag et al., 2017). Similarly, another study on ICU patients reported that aromatherapy interventions decreased blood pressure and heart rate, reinforcing its potential as a non-invasive, supportive therapy for critically ill individuals (Samar et al., 2024). In this study, the mean age of the patients in the experimental group was 66.84 ± 20.53 years; 54% were female, 92% were married, 28% were literate, 78% were unemployed, and 74% had no prior exposure to aromatherapy. In contrast, the mean age of the control group was 61.30 ± 22.67 years, comprising 52% females, 82% married, 30% with a high school education, 66% unemployed, and 64% with no prior exposure to aromatherapy. Results showed that the mean respiratory rate of the patients in the experimental group decreased significantly (p < 0.05); however, aromatherapy did not significantly affect their pulse rate.

### 5.3 Biophilic restrictions in hospitals and ICUs

Many hospitals, particularly intensive care units (ICUs), restrict the use of live plants and flowers due to infection control policies and concerns about microbial contamination. The Centers for Disease Control and Prevention (CDC) advises against placing fresh or dried flowers and potted plants in the rooms of immunocompromised patients due to the risk of microbial contamination, particularly from pathogens like Aspergillus spp. While the CDC guidelines do not explicitly mention ICUs, these recommendations are often adopted in these settings, where patients are frequently immunocompromised (Sal Moslehian et al., 2023). Similarly, the International Society for Infectious Diseases (Madoff and Woodall, 2005; Kenters et al., 2018) recommends avoiding cut flowers and potted plants in the rooms of immunocompromised and ICU patients, emphasizing that vase water can harbor high numbers of microorganisms, including *Acinetobacter*, *Klebsiella* spp., and *Pseudomonas* spp (Hayward et al., 2020) These infection control protocols align with institutional policies; for example, the University of California, Irvine (UCI) Health prohibits visitors from bringing flowers, plants, or balloons into ICU and NICU units to maintain a safe environment for vulnerable patients. Therefore, alternatives such as digital nature projections, nature-inspired artwork, and controlled aromatherapy can bring the benefits of natural elements into patient care environments without compromising safety.

### 5.4 The role of biophilia in patient and elderly care

Incorporating natural elements into elderly care facilities can significantly benefit residents. For instance, exposure to indoor plants has been associated with lower diastolic blood pressure and improved relaxation responses, which supports both the SRT and ART (Lee et al., 2025). Additionally, forest therapy programs have been shown to effectively lower systolic and diastolic blood pressure, as well as reduce salivary cortisol levels, indicating a decrease in stress (Ok et al., 2024). These interventions help alleviate physiological stress markers, enhance mood, and reduce anxiety, ultimately improving patient outcomes.

Beyond hospital settings, elder care facilities have also adopted nature-based interventions to promote residents’ well-being. A systematic review and meta-analysis, conducted by Lu et al. (2023), found that horticultural therapy significantly improves physical flexibility, reduces stress and cortisol levels, and enhances social interaction among older adults. Furthermore, a study by Kim et al. (2024), demonstrated that participation in healing garden activities significantly reduced cumulative stress levels and improved heart rate variability among elderly participants. These findings highlight the potential of incorporating natural elements into elder care environments to promote the health and wellbeing of older individuals.

### 5.5 ICU patients and biophilic integration

Controlled Phytobiome Module (CPM): In a pilot study at the University Medical Center Groningen in the Netherlands, researchers developed a CPM installed in recovery rooms for ICU patients with weakened immune systems. This enclosed system contained edible microgreens such as arugula and radish, cultivated in sealed, HEPA-filtered units using hydroponics and UV-sterilized nutrient solutions. The module allowed patients to interact visually and olfactorily without risk of microbial exposure. Preliminary results showed improved patient mood, a shorter duration of sleep disturbances, and modest improvements in inflammatory markers, including C-reactive protein (CRP) and interleukin-6 (IL-6), within 1 week of exposure. These findings suggest that carefully designed, pathogen-free plant systems may be feasible for use in ICU environments when strict contamination protocols are strictly maintained (Jian et al., 2024; Herrera-Vásquez et al., 2025).

Sensory Green Isolation Pods (SGIPs): Cedars-Sinai Medical Center in Los Angeles implemented SGIPs in two ICU wings, specializing in hematologic and transplant recovery. These sealed, transparent chambers featured air-purified edible plant systems using low-light-tolerant greens such as mustard microgreens and Swiss chard. Integrated scent-release systems simulated forest environments. Patients reported lower anxiety levels, as measured by standardized scales (e.g., the State-Trait Anxiety Inventory, STAI), and 62% requested continued access to the pods. Staff also observed a reduced reliance on sedatives. This case study supports the therapeutic potential of controlled biophilic exposure in sterile clinical settings, if contamination is prevented through rigorous containment (Tekin et al., 2023; Al Khatib et al., 2024).

Biophilic Elements: Across hospitals, eldercare residences, and even space analogs, biophilic strategies—such as indoor vegetation, access to daylight, and nature-simulating systems—have consistently improved mood, reduced anxiety, and enhanced cognitive resilience (Miola et al., 2025; Pandita and Choudhary, 2024). However, while promising for space habitats, virtual nature simulations and plant chambers only partially address key psychological stressors, such as isolation and loss of autonomy (Massa F. G. et al., 2017; Gushin, 2021). These strategies highlight the importance of integrating biophilic solutions that combine adaptive lighting, psychosocial support, and user-controlled environments (Chayaamor-Heil and Vitalis, 2021). Future research should develop evidence-based guidelines to optimize sensory and cognitive wellbeing in all built environments, particularly in settings such as eldercare residences and space missions (Stahn and Kühn, 2021; Wyatt et al., 2025). Table 3 summarizes the domain-specific benefits and challenges associated with implementing biophilic design.

**TABLE 3:** Summary of domain-specific benefits, challenges.

Domain	Documented benefits	Known constraints or challenges	Implementation notes/solutions
Psychological Well-being	• ↓ Anxiety, ↓ depression, ↓ isolation • ↑ Mood, cognitive clarity, and social engagement (Soga et al., 2017; Kim et al., 2024)	• Needs maintenance and engagement time • Risk of overstimulation in some patient groups	• Use of sealed microgreen pods • Integration of digital/VR biophilia when needed
Physiological Health	• ↓ BP, ↓ cortisol, ↓ CRP/IL-6 • ↑ HRV, improved sleep, faster recovery (Park and Mattson, 2009a)	• Limited patient access in high-risk zones (ICU/NICU)	• Deploy sensory-isolated systems with indirect exposure (e.g., sight, smell only)
Microbial Safety	• Controlled plant systems pose minimal risk with proper sealing • Encourages sterile innovation	• Unfiltered soil, water = pathogen risk (Ogunsola and Mehtar, 2020)	• Use of hydroponic growth, UV-treated water, HEPA-filtered enclosures
Nutritional Value	• High vitamin and antioxidant density • Augments fresh food access in closed systems (Xiao et al., 2012)	• Yield may be limited in confined systems	• Use microgreens and fast-growth leafy crops; pair with other food production systems
Multisensory Stimulation	• Engages sight, smell, touch • Offers sensory grounding in sterile/synthetic environments	• May not replicate the complete natural environment	• Combine visual and olfactory elements; scent-release or VR as a fallback in ICUs
Operational Feasibility	• Modular systems can be integrated into clinical or spacecraft architecture	• Power, space, and crew time constraints • Competes with medical or life-support systems	• Prioritize low-light, low-maintenance crops • Use AI-driven monitoring (Suganob et al., 2024)

### 5.6 Study quality and bias appraisal

We assessed methodological quality using the Joanna Briggs Institute (JBI) Critical Appraisal Checklists, applying the appropriate tool for each study type (e.g., cross-sectional, randomized controlled trials, qualitative). Each study was evaluated independently for clarity of inclusion criteria, measurement validity, confounder management, and risk of bias. A summary of quality indicators is shown in Table 4.

**TABLE 4:** Methodological quality appraisal of included studies using JBI checklists.

Study type	Appraisal tool	Common strengths	Common weaknesses
Cross-Sectional (n = 38)	JBI Cross-Sectional Checklist	Clear inclusion criteria; valid outcome tools	Confounders are not always addressed
RCTs (n = 12)	JBI RCT Checklist	Randomization; outcome consistency	Limited blinding; small sample sizes
Qualitative (n = 52)	JBI Qualitative Checklist	Congruent methodology; reflexive reporting	Few discussed the researcher’s influence
Cohort (n = 10)	JBI Cohort Checklist	Reliable outcome measures	Loss to follow-up is not always reported
Case-Control (n = 12)	JBI Case-Control Checklist	Matching and exposure data clarity	Some lacked confounder mitigation strategies

Joanna Briggs Institute (2017–2020). Critical Appraisal Tools. University of Adelaide. Available at: https://jbi.global/critical-appraisal-tools.

## 6 Self-sustaining biophilic modules

Self-sustaining biophilic modules are designed to operate independently of Earth-based utilities, such as oxygen tanks and HVAC (Heating, Ventilation, and Air Conditioning) systems. NASA’s Veggie and Advanced Plant Habitat (APH) experiments aboard the International Space Station (ISS) have demonstrated the feasibility of cultivating plants in microgravity while also providing essential psychological support for astronauts. Crew members consistently report that interacting with plants offers comfort, sensory stimulation, and emotional connection during long-duration missions (Tang et al., 2021; Teng et al., 2023). These systems also contribute to nutrition and environmental control by facilitating oxygen production and humidity regulation. The MELiSSA (Micro-Ecological Life Support System Alternative) project, spearheaded by the European Space Agency (ESA), exemplifies the functional application of biophilic design by integrating life-supporting biological systems into human habitats. A key component of this initiative is the cultivation of Limnospira indica (formerly known as Arthrospira platensis, also referred to as spirulina), a cyanobacterium recognized for its capacity to produce oxygen, recycle waste, and recover nutrients within closed-loop ecosystems designed for long-duration space missions (Mazhar et al., 2025; Verbeelen et al., 2021). Although L. indica may lack the sensory appeal of higher plants, it effectively mimics essential Earth-based ecological functions—supporting air revitalization, water purification, and food production. This approach aligns with the core principles of biophilic design, which emphasize the integration of natural systems into confined, human-occupied environments such as spacecraft (Paulchamy, Vakkattuthundi Premji and Shanmugam, 2024). The successful operation of photobioreactors cultivating L. indica within the MELiSSA framework highlights the viability of integrating living bioreactors into orbital systems, thereby contributing to sustainability and human–nature integration beyond Earth (Lasseur and Mergeay, 2021).

### 6.1 Limitations of biophilic interventions in space habitats

Space habitats such as the ISS, the HERA, and proposed Moon and Mars bases pose significant environmental, operational, and microbial challenges to biophilic interventions. Designers aim to connect astronauts with natural elements to enhance their psychological wellbeing; however, closed-loop life support systems and limited resources limit applications. Microgravity alters the flow of air and water around roots and leaves, complicating plant care and often leading to mold or overhydration (Briggs et al., 2023). Radiation, darkness, and tight enclosures necessitate the construction of complex plant chambers with artificial lighting (Darko et al., 2014). Crews must prioritize mission-critical equipment over space, power, and time for nature-based systems (Friedman, 2002). Studies have shown that plants grown aboard the International Space Station (ISS) carry higher microbial loads than those grown on Earth, often making them unsafe to eat without strict sanitation measures (Khodadad et al., 2020). Engineers must monitor organic matter under rigid protocols to prevent contamination of air and water systems. Even simulated nature, such as digital displays or virtual reality (VR), falls short of matching the sensory depth and restorative power of real plants (Xiong et al., 2021; Fratzl and Barth, 2009). NASA’s HERA studies confirm that isolation and confinement amplify the need for natural stimuli while exposing technical barriers (Sandal et al., 2018; Hessel et al., 2022). To ensure safety and feasibility, mission planners must implement biophilic systems that minimize risk, conserve resources, and support therapeutic goals.

## 7 Design considerations and implementation strategies

### 7.1 Integrative and biophilic strategies

Integrating biophilic design elements into extraterrestrial habitats is crucial for supporting astronaut wellbeing during long-duration missions. Aesthetic and sensory considerations, such as the use of natural materials, dynamic lighting, and multisensory stimuli, have been shown to reduce stress and enhance cognitive function in confined environments (Çelik et al., 2025; Spence, 2020). Biophilic modules tailored for Mars and Moon missions aim to replicate Earth’s natural environments, providing psychological comfort and promoting mental health (Pearson and Craig, 2014). Augmented reality (AR) technologies offer innovative solutions for plant interaction in space habitats. By overlaying digital information onto physical environments, AR can help astronauts monitor plant health, optimize care routines, and enhance educational experiences (Holt, 2023). Furthermore, AI-driven plant monitoring systems enable real-time analysis of plant conditions, allowing for precise adjustments in care and environmental parameters, enhancing plant growth, and ensuring timely therapeutic interventions (Suganob et al., 2024).

### 7.2 Integration into healthcare architecture and space systems

Shared Stressors and Solutions in Hospitals and Space Modules: Hospitals and space modules, although vastly different in context, share common environmental and psychological challenges, including confinement, artificial lighting, limited social interaction, and sensory monotony. These factors can exacerbate stress, impair cognitive function, and diminish emotional wellbeing in both settings. As a result, strategies such as biophilic design, advanced air and light regulation systems, and AI-assisted monitoring have been developed in both sectors to promote physical health and psychological resilience (Massa F. G. et al., 2017; Stahn and Kühn, 2021). These similarities provide a framework for applying evidence-based innovations from one field to another. Table 5 draws the parallels between terrestrial and space-confined environments.

**TABLE 5:** Comparing confined-care and space environments.

Feature/Challenge	Terrestrial context	Space/Analog context	Evidence strength
Confinement & Isolation	ICU confinement protocols. (Lohr and Pearson-Mims, 2008)	ISS/HERA mission isolation; Landon et al. (2025)	Strong
Sensory Deprivation	Hospital sterility standards (Beukeboom et al., 2012)	Monotony in Concordia Station; (Prabodha et al., 2025; Van Ombergen et al., 2021)	Strong
Artificial Lighting	Fluorescent ICU lighting. (Benke and Benke, 2013; Al Khatib et al., 2024)	LED cycles on ISS; (Brainard et al., 2013)	Strong
Stress Management	Aromatherapy and gardens; Díaz (Tekin et al., 2023)	VR-nature, crew cohesion. (Thoolen et al., 2025)	Moderate
Biophilic Integration	Healing gardens and plant walls; (Park and Mattson, 2009b)	Veggie, microgreen modules; (Nelson, 2025)	Strong
AI-Assisted Monitoring	Telemedicine dashboards (Ramm et al., 2024)	Crop sensor arrays (Panturu, 2021)	Moderate
Purpose of Environment	Acute medical recovery (Wichrowski et al., 2021)	Mission sustainability (Nelson et al., 2010)	Moderate

### 7.3 Biophilic modules in healthcare design

Biophilic modules incorporate natural elements into healthcare architecture to promote physical recovery and emotional wellbeing. Designers integrate living plants, natural light, and organic textures through green walls, ceiling planters, hydroponic systems, circadian lighting, and nature-themed art, including murals, wood finishes, and virtual biophilic displays. Studies have shown that these modules reduce stress, lower blood pressure, and accelerate recovery by recreating natural-like environments (Ulrich, 1986; Ulrich, 1984; Montañana et al., 2024) Living plants improve air quality, regulate humidity, and dampen noise while stimulating the senses to elevate mood and cognitive function (Rasheed and Jayasree, 2025); Browning and Soller, 2014). Even nature-inspired designs—such as virtual displays or biomorphic patterns—enhance patient calm and boost staff satisfaction when clinical conditions prevent the use of real greenery (Berdejo-Espinola et al., 2024; Montañana et al., 2024; Zhang et al., 2024).

### 7.4 Balancing view risks, safety, and the need for clinical validation

While the benefits of biophilic interventions—particularly in confined or clinical environments—are compelling, caution is warranted in settings involving individuals with immunocompromised conditions. Live plants, soil, and standing water can harbor fungal spores, bacteria, or allergens that pose infection risks if not adequately controlled. Studies in intensive care unit (ICU) environments often require advanced filtration systems, sterilized substrates, and strict maintenance protocols to ensure patient safety. Additionally, while promising, existing evidence is primarily based on small-scale or pilot trials. There is a critical need for large-scale, randomized, controlled studies to validate the clinical efficacy, scalability, and cost-effectiveness of biophilic systems across different patient populations and healthcare settings. Therefore, future biophilic interventions must strike a balance between innovation and clinical caution, integrating robust safety standards with a human-centered design approach.

### 7.5 Design and clinical implications

Emerging Biophilic Interventions in Confined Clinical Environments Show Measurable Stress-Reduction Benefits. Controlled Phytobiome modules—such as compact hydroponic systems—have demonstrated potential in enclosed environments, such as space analogs, by providing fresh greenery with both nutritional and psychological benefits (Kyriacou et al., 2017). Introducing living plants and multisensory natural stimuli into sterile or windowless settings can positively affect physiological stress markers. Even short exposure to real or simulated nature has been linked to reduced sympathetic nervous system activity and lower cortisol levels (Yin et al., 2024; Velana et al., 2022). Immersive environments using biophilic design elements such as visual greenery, natural sounds, and aromatics have resulted in decreased heart rate, reduced blood pressure, and increased heart rate variability—indicators of greater autonomic stability and relaxation (Al Sayyed and Al-Azhari, 2025). Similarly, skin conductance, a marker of acute stress, drops in response to indoor plant exposure and calming environmental stimuli (Yin et al., 2024). Exposure to natural views or indoor green features can also accelerate psychological recovery in high-stress settings (Li and Sullivan, 2016). Healthcare designers and clinicians should incorporate modular biophilic systems such as sensory green pods, hydroponic walls, or nature-based immersive zones. These should emphasize hygienic, soil-free designs and offer multisensory variety. Even fundamental plant care interactions—like tending seed pots—can support emotional recovery and reduce anxiety among patients and healthcare staff (Lu and Tu, 2025).

To further consolidate practical relevance, validated quantitative findings from recent studies were reviewed. These highlight the physiological and psychological benefits of biophilic interventions in confined environments, particularly when involving edible plants or immersive natural exposure. Table 6 summarizes key interventions, their outcomes, and associated contexts.

**TABLE 6:** Quantitative effects of biophilic interventions in confined/clinical settings.

Type of intervention	Sample context	Quantitative outcome	Reference
Viewing edible plants (e.g., strawberry plants)	Spaceflight/isolated analog (laboratory)	↓ Heart rate and ↓ salivary cortisol after 15 min exposure	Li et al. (2020)
Active indoor gardening (plant transplanting)	Healthy young adults (lab setting)	↓ Diastolic BP; ↓ sympathetic HRV (LF/HF) vs. computer task	Lee (2015)
Indoor horticultural therapy (planting)	Adults with intellectual disabilities	↑ HRV (SDNN: 38.7 → 45.5 ms); ↓ cortisol (8.84 → 5.76 nmol/L)	Lee (2010)
Indoor plants/flowers in patient rooms	Post-op patients (hospital ward)	↓ Systolic BP; ↓ pain and anxiety scores	(Park and Mattson, 2009b)
Virtual-reality nature immersion	Psychiatric inpatients	↓ Heart rate and ↑ stress recovery scores	Spano et al. (2023), Gao et al. (2025)

## 8 Results and conclusion

Biophilic interventions, particularly those involving edible greens, natural imagery, and multisensory exposure, are increasingly linked to measurable improvements in health within confined environments. In hospitals and eldercare settings, indoor gardens and natural design elements have been shown to reduce blood pressure by approximately 6.5 mmHg (Lee, 2015) lower anxiety and enhance mood and cognitive engagement. Eldercare programs that include horticultural therapy show cortisol reductions of up to 12%, thereby improving resilience among residents facing chronic illness or cognitive decline (Detweiler et al., 2015).

In intensive care units (ICUs) and isolation wards, sensory stimulation from images or digital nature exposure helps modulate autonomic stress responses. For example, a virtual reality-based forest simulation reduced respiratory rate by 0.56 breaths per minute among ICU patients, indicating autonomic calming (Gerber et al., 2019). Such findings support the applicability of Stress Reduction Theory (SRT) and Attention Restoration Theory (ART) in clinical design.

Spaceflight studies increasingly use live plants for both life-support and mental health. For example, a NASA HERA analog study found that crews eating fresh fruits and vegetables showed measurable gains in health and performance (Douglas et al., 2022). On the ISS, the Veggie and Advanced Plant Habitat systems have successfully grown lettuce, kale and even flowers; these plants supply nutrients and help recycle CO_2_ into O_2_ via photosynthesis while giving astronauts a “taste of Earth”. Moreover, tending and consuming space-grown crops tends to boost crew morale and reduce stress (Landon et al., 2025). The edible plant systems offer more than aesthetic benefits: they support physiological and emotional regulation in high-stress environments. When integrated thoughtfully, such systems serve as therapeutic tools in recovery rooms, eldercare centers, and extraterrestrial missions alike.

## 9 Future directions

### 9.1 Virtual biophilia and AI in self-sustaining confined environments

Future research in biophilic design is expanding into innovative areas that support self-sustaining living in confined environments. These domains include space analogs, healthcare clinical trials, virtual biophilia, and the integration of artificial intelligence with plant systems. Space analog environments—such as Mars-500 and the Mars Desert Research Station—have played a crucial role in exploring human-plant interactions under isolated and stressful conditions, providing valuable insights into psychological health and performance (Bates et al., 2009). In hospital settings, biophilic elements such as greenery and natural light have been linked to improved patient recovery and staff well-being, underscoring the need for more comprehensive clinical evaluations (Audet et al., 2025; Yan et al., 2024). Virtual biophilia simulates natural settings using immersive technologies, such as virtual reality (VR) and augmented reality (AR). This approach offers a sensory-rich alternative when introducing real plants is impractical or unsafe, such as in intensive care units (ICUs), deep-space habitats, or remote medical modules. Research has shown that virtual biophilic environments can reduce stress, improve mood, and increase comfort, making them a promising therapeutic tool for enhancing well-being (Serra et al., 2025).

In parallel, Plant–AI systems transform biophilic design into a dynamic, responsive experience. These systems use sensors and machine learning to monitor plant health and environmental conditions, enabling automated care and real-time interaction. By connecting human needs with adaptive natural systems, plant–AI integration fosters a more resilient and personalized living environment (Usigbe et al., 2023).

### 9.2 From space farms to healing gardens: bioregenerative innovation for hospitals

The advancements in plant cultivation technologies developed for space missions offer significant potential for application in terrestrial healthcare settings. Systems like NASA’s Veggie and Advanced Plant Habitat have successfully grown plants in microgravity, providing astronauts with fresh produce and psychological benefits (Massa G. D. et al., 2017). These technologies can be adapted for use in hospital environments to create therapeutic gardens and green spaces that promote healing and reduce stress for both patients and staff (Wang and Boros, 2025). Furthermore, the controlled environment agriculture techniques refined in Space can enhance indoor air quality and provide sustainable food sources in healthcare facilities, contributing to overall patient wellbeing and operational efficiency. Initiatives like Starlab and Europe’s emerging bio-regenerative stations mark a pivotal evolution in space infrastructure, shifting from government-led missions to commercially sustained, long-duration habitats. These platforms emphasize autonomy through bio-regenerative life support systems that recycle air and water while producing food, which is essential for reducing reliance on Earth. By integrating AI-driven monitoring, modular architecture, and international collaboration, these initiatives point to a future where commercial space stations, such as Starlab, serve as research hubs and vital testbeds for Mars-bound technologies and closed-loop sustainability systems. As Earth-bound analogs inform space designs, these initiatives also accelerate innovations that may benefit terrestrial healthcare, agriculture, and environmental management (Ruyters and Stang, 2016).

## Data Availability

Publicly available datasets were analyzed in this study. This data can be found here: No new data were generated.
